# A decade of *Genome Medicine:* toward precision medicine

**DOI:** 10.1186/s13073-019-0624-z

**Published:** 2019-02-28

**Authors:** Rabia Begum

**Affiliations:** Genome Medicine, London, UK

Decoding the human genome in the context of the molecular mechanisms of disease, disease staging, disease progression, and patient outcome has been fundamental to research and practice in genomic medicine. This evolving field has seen significant strides in recent years with advances across a range of disciplines including: the precision editing of the microbiome to manage dysbiosis-associated inflammation [1]; the first-in-human application of a personalized neoantigen vaccine in melanoma patients [2]; FDA approval of pembrolizumab for mismatch repair-deficient or microsatellite-instable high-solid tumors, irrespective of tumor type [3]; and gene therapy in patients with a severe form of β-thalassemia, which can reduce or eliminate lifelong transfusion dependency [4].

2019 marks the 10th anniversary of the launch of *Genome Medicine*, which serves as a global platform bridging basic science and clinical research in human health and disease. The journal’s scope encompasses all areas in the application of genetics, genomics, multi-omics, and high-throughput technologies, and it serves a community who are working to understand, diagnose, and treat disease. To celebrate our 10th anniversary, we have selected twenty of our most notable articles of the past decade [5] that have shaped the landscape of human health and disease research. Although studies that make headway in terms of their clinical impact and practice are of paramount importance, the basic science that is published in the journal remains the keystone for understanding disease mechanisms, risk prediction, and therapeutic strategies. *Genome Medicine*’s broad scope allows us to capture the most exciting advances across multidisciplinary fields, enabling the inclusion of evolving areas such as the integration of imaging or histopathology data with machine learning or artificial intelligence. The field of genome medicine is much more than searching for answers at the genomic level; it is inherently integrated with a multi-data, multi-layer, systems level approach at high resolution that has impact on clinical practice and care at its core.

Precision medicine has organically become an increasingly important focus of the journal, meeting the community’s need for the rapid dissemination of advances that have the potential to change fundamentally how healthcare is practiced. For example, Kung and colleagues [6] describe the integration of clinical next-generation sequencing to provide definitive diagnoses in the pediatric hematology-oncology practice of the Precision in Pediatric Sequencing Program. Bedard and colleagues [7] show that in two prospective studies, the Integrated Molecular Profiling in Advanced Centers Trial (IMPACT) and the Community Molecular Profiling in Advanced Cancers Trial (COMPACT), genotype-matched clinical trials using the molecular profiling of advanced solid tumors is associated with increased objective tumor response rate. These attempts to personalize patient care have been integrated with electronic health records, which are increasingly being used to combine genomic and phenotypic data within the healthcare system. Treatment decisions can be guided by assessment of the landscape of tumor mutational burden; for example, using comprehensive genomic profiling of > 100,000 patient tumors, Frampton and colleagues were able to identify mutation-rich tumors, supporting rational expansion of the patient population that could benefit from immunotherapy [8].

Although the past decade has seen the rapid evolution of the toolbox for genomic technologies [9–13], controversies have inevitably arisen. Reports of CRISPR-Cas9 germline genome editing in human embryos have been met with widespread ethical concerns and have re-ignited discussions on the governance of genome editing research in clinical applications.

Progress in the GWAS discovery field has provided us with remarkable insights into complex trait genetics and disease biology [14]. It has also facilitated the use of polygenic risk scores (PRSs), which summarize genome-wide genotype data into a single variable that measures genetic liability with respect to a certain trait or disease [15]. Recent work has demonstrated that PRSs can identify disease risk in individuals with accuracy that is comparable to that conferred by the presence of rare monogenic mutations [16], showing that PRSs can indeed capture actionable risk information. Nevertheless, there remain controversies surrounding the clinical utility of PRSs, around whether PRSs can capture clinical heterogeneity, and associated with the potential bias that may result from the lack of diverse ancestry population data.

Open science in medical research with respect to unrestricted access and data sharing has been limited, but *Genome Medicine* has been at the leading edge of open access in the genomic medicine space. This is underscored by our position on data sharing, a practice that (where compliant with ethical approvals) is indispensable for accelerated research progress, particularly in the clinical trial setting. In this context, the ongoing debate about the definition of consent has intensified in recent years with discussions around what a participant is consenting to, and about whether the data rights are transferred to researchers or whether data ownership remains with the participant who authorized it for a defined research use [17]. What does that mean for re-analyses of data in the public domain that have been de-identified? Can these data be re-identified with additional layers of re-analyses? Data access and security is thus a pressing concern in medical research. Discussion is needed around how data use can be better regulated and how data access can be managed for specific data types; for example, how can clinical trial data be managed in a way that both respects the terms under which the participants consented and ensures equity in data use [18]?

*Genome Medicine* seeks to engage with communities from all of these research areas, including those studying the microbiome, infectious disease, metabolic disease, cardiology, and neurogenomics. We remain interested in advances at the patient and population levels that will refine our understanding of healthy, at-risk, and disease states. 2019 and beyond will see the journal focus on interpreting variants of uncertain significance, on the clinical interpretation of genome variation, and increasingly on clinical trials, both interventional and observational, with the goal of moving toward precision medicine. We will continue to support multidisciplinary and open research, drawing together basic science and clinical research communities to set standards that will inform research design and future clinical practice. We would like to express our deepest gratitude to our editorial board, section editors, guest editors, authors, reviewers, and readers who have helped to shape the journal over the past decade. We look forward to the next ten years of *Genome Medicine* and to working with you in this evolving landscape of medical research.

## Abbreviation

PRS: Polygenic risk score
